# Efficacy of Autogenous Bone Marrow Aspirate as a Fusion-promoting Adjunct to Anterior Cervical Discectomy and Fusion: A Single Center Retrospective Cohort Study

**DOI:** 10.7759/cureus.2636

**Published:** 2018-05-16

**Authors:** Sean M Barber, Majdi Radaideh, Rob Parrish

**Affiliations:** 1 Department of Neurosurgery, Houston Methodist Neurological Institute, Houston, USA; 2 Neuroradiology, Houston Methodist Neurological Institute, Houston, USA

**Keywords:** spinal fusion, bone marrow, autograft

## Abstract

Background

Autogenous iliac crest bone marrow aspirate (BMA) has been shown to be a safe osteobiological adjunct to anterior cervical discectomy and fusion (ACDF), but little evidence exists to support its superiority to traditional methods. The object of this study was to retrospectively evaluate two cohorts of patients undergoing ACDF – with or without the use of BMA – in an effort to better characterize the clinical and radiographic outcomes associated with the use of BMA in ACDF.

Methods

The charts of all patients undergoing ACDF with a collagen-hydroxyapatite (CHA) sponge, local vertebral autograft and a polyetheretherketone (PEEK) interbody graft with or without BMA by a single staff neurosurgeon between 2011 and 2016 were retrospectively reviewed. Post-operative dynamic plain films and CT scans for each patient were reviewed and each instrumented level was independently evaluated for fusion over time.

Results

A total of 203 cervical levels were instrumented in 92 patients (with BMA, 52 patients, 122 levels; without BMA, 40 patients, 81 levels). The mean radiographic follow-up period was 21.4 ± 18.4 months, over which time 154 of 203 (75.6%) instrumented cervical levels were found to have fused (BMA group, 93/122 segments fused [76.2%]; non-BMA group, 61/81 segments fused [75.3%], p = 1). Kaplan-Meier survival analysis demonstrated a higher probability of fusion at any given time point for the BMA group when compared with the non-BMA group (p < 0.001, log-rank test).

Conclusions

BMA is a readily accessible, low-cost adjunct to ACDF that enhances the fusion rates seen with a CHA/PEEK allograft combination.

## Introduction

Although anterior cervical discectomy and fusion (ACDF) is typically successful at producing osseous fusion of instrumented levels, pseudarthroses – when present – may necessitate re-operation and/or serve as a source of ongoing pain and neurologic symptoms. Efforts to reduce the pseudarthrosis rate after ACDF have led to procedural refinements such as anterior plating systems [1] and the use of a wide variety of grafting materials and osteobiologic adjuncts [2,3]. While autogenous tricortical iliac crest bone graft (ICBG) has been used for decades as an effective graft material for ACDF and is often considered the “gold standard” [2], the morbidity associated with ICBG harvesting has led many surgeons to consider alternative grafting materials [4]. Because the osteogenic potential of cancellous bone has been shown to originate from the osteoprogenitor cells present within bone marrow, many have advocated for the use of bone marrow aspirate as an adjunct to fusion procedures within the spine and elsewhere [5-9]. Although the use of bone marrow aspirate (BMA) as an adjunct to ACDF has been shown to be relatively safe and outcomes have been shown to be relatively favorable [8,10], definitive evidence of efficacy and/or superiority to other methods is lacking [11].

The authors performed a retrospective review of patients undergoing ACDF – with or without the use of BMA - by a single neurosurgeon at our institution over a period of five years in order to better characterize the efficacy of BMA as a fusion-promoting adjunct to ACDF.

## Materials and methods

Study design

This single-center, retrospective cohort study was carried out with the approval of the Institutional Review Board of our hospital. Charts for all patients having undergone ACDF by the senior author at our institution between April 2011 and May 2016 were reviewed. The senior author did not use BMA as an adjunct to ACDF initially during the study period, but began to do so semi-exclusively starting in 2013.

Patient identification took place through review of institutional operative records. All patients having undergone ACDF by the senior author between April 2011 and May 2016 were considered potentially eligible. Exclusion criteria included: (1) history of prior ACDF at a given segment, (2) corpectomy at a given segment, (3) absence of at least six months of radiographic (i.e., dynamic cervical spine plain films and/or cervical spine computed tomography (CT) scans) and clinical follow-up post-operatively, (4) lack of access to pre-operative films, or (5) simultaneous posterior decompression and/or instrumentation. Clinical data collected included operative complications, post-operative clinical status, and need for re-operation. Radiographic assessment of fusion was based on cervical flexion-extension x-rays and/or CT scans.

Surgical technique

ACDF was performed in each case using standard techniques. In patients undergoing bone marrow aspiration, however, the ipsilateral anterior iliac crest is also prepared and draped. After anterior cervical discectomy is complete, a small incision is then made above the anterior superior iliac spine. An 11G bone marrow needle and trocar are inserted into this incision and carried down to the bone. The needle and trocar are then advanced 2 cm in line with the slope of the anterior iliac crest. The trocar is then removed and a 2 mL of bone marrow is aspirated. In cases where additional BMA is required (e.g., multi-level fusions), the needle is then advanced slightly before withdrawing additional BMA. The BMA is then passed through a collagen-hydroxyapatite (CHA) sponge within a vacuum chamber without centrifugation or other alterations. The treated sponge is then placed within the pre-selected polyetheretherketone (PEEK) graft along with local vertebral autograft prior to graft implantation. Additional sponge material is placed laterally near the uncovertebral joints at the instrumented level. The procedure for patients undergoing ACDF without the use of BMA was identical, with the exception that BMA was not harvested, and the CHA sponge was instead soaked with normal saline prior to implantation.

Criteria for determination of radiographic fusion

Clinical and radiographic follow-up was maintained for at least six months post-operatively in all patients. Radiographic fusion was determined after review of the post-operative films with a blinded neuroradiologist. Each instrumented segment was evaluated independently. In order to be considered radiographically fused, each of the following criteria had to be satisfied at the instrumented segment: (1) less than 1 mm interspinous movement on flexion/extension films, (2) less than 2 degrees of angular movement of instrumented endplates on flexion/extension films, (3) absence of interfacet movement on flexion/extension films, and (4) absence of radiolucencies around the graft or other hardware. If CT was available, evidence of bridging trabecular bone at the instrumented level was required for the segment to be considered fused. If any of these criteria were not satisfied, the segment was deemed unfused.

Statistical methods

Frequency distributions and summary statistics were calculated for all clinical and radiographic variables. Fisher’s exact test was used to compare distributions for categorical variables and t-tests were used to investigate differences in the distributions of continuous variables between subsets of patients classified by dichotomous data. Pearson correlation coefficients were calculated to evaluate associations between fusion rates and dichotomous patient data (e.g., age, gender, smoking status). Kaplan-Meier survival analysis was used to compare the probability of fusion over time for segments in each of the two study groups. A p-value of 0.05 or less was considered significant.

## Results

Participants

Ninety-two patients and 203 operative levels were included in this retrospective review. Fifty-two of the included patients (122 instrumented levels) underwent ACDF with the use of autogenous iliac crest BMA, while the other 40 patients (81 instrumented levels) underwent ACDF without BMA. Fifty-six of the study patients were female (60.9%) and 36 patients were male (39.1%). The mean age for the entire cohort was 53 ± 10 years. Active smoking status was confirmed by chart review in 14 patients (15.2%). Study patients presented with some combination of neck pain (N = 63, 68.5%), radiculopathy (N = 53, 57.8%) and/or myelopathy (N = 34, 37%). Patient demographics and pre-operative symptoms are detailed in Table 1.

**Table 1:** Demographics, presenting symptoms and comorbidities. Demographics, presenting symptoms and comorbidities of 92 patients undergoing ACDF with – or without – the use of autogenous BMA. ACDF: Anterior cervical discectomy and fusion; BMA: Bone marrow aspirate; HTN: Hypertension; HLD: Hyperlipidemia; DM: Diabetes mellitus; CAD: Coronary artery disease; RA: Rheumatoid arthritis; Afib: Atrial fibrillation.

	Total	BMA	Non-BMA	p
N	92	52	40	
Segments Instrumented	203	122	81	
Segments Instrumented per Patient	2.21	2.35	2.03	0.062
Age	53	54	53	0.66
Male	39 (39.1%)	18 (34.6%)	18 (45%)	0.171
Female	56 (60.9%)	34 (65.3%)	22 (55%)	0.171
Current Smoker	14 (15.2%)	5 (9.6%)	9 (22.5%)	0.557
Pre-operative Symptoms				
Neck Pain	63/92 (68.5%)	39/52 (75%)	24/40 (60%)	0.174
Radiculopathy	53/92 (57.6%)	29/52 (55.8%)	24/40 (60%)	0.832
Myelopathy	34/92 (37%)	19/52 (36.5%)	15/40 (37.5%)	1
Comorbidities				
Hypertension	35/92 (38%)	20/52 (38.5%)	15/40 (37.4%)	1
Hyperlipidemia	18/92 (19.6%)	5/52 (9.6%)	13/40 (32.5%)	0.008
Hypothyroidism	15/92 (16.3%)	9/52 (17.3%)	6/40 (15%)	1
Diabetes Mellitus	9/92 (9.8%)	4/52 (7.7%)	5/40 (12.5%)	0.495
Coronary Artery Disease	8/92 (8.7%)	3/52 (5.8%)	5/40 (12.5%)	0.287
Rheumatoid Arthritis	6/92 (6.5%)	2/52 (3.8%)	4/40 (10%)	0.398
Atrial Fibrillation	5/92 (5.4%)	1/52 (1.9%)	4/40 (10%)	0.163

Clinical outcomes

The mean clinical follow-up length for the entire study population was 22.5 ± 18.4 months. 82/92 (89.1%) patients reported improvement in their pre-operative symptoms at the latest follow-up visit, while 10/92 patients reported stable symptoms (10.9%). Minor complications occurred in 4/92 patients (4.3%), including dysphagia (2/92 patients, 2.2%), dysphonia (1/92 patients, 1.1%), and Horner’s syndrome (1/92 patients, 1.1%). No patient underwent revision of their ACDF during the study period, but 8/92 patients (8.7%) underwent further decompression at the same cervical segment from a posterior approach due to persistent or worsening neurological symptoms and persistent spinal cord or nerve root compression on post-operative imaging. No patient reported prolonged pain at the BMA donor site. Several patients (9/92 patients, 9.8%) were placed on an osteostimulator device post-operatively due to less-than-satisfactory bone growth on post-operative follow-up plain films. Post-operative symptom improvement (P = 0.096), complication rates (P = 0.314), re-operation rates (P = 0.724) and rates of osteostimulator use (P = 0.495) were not statistically different between BMA and non-BMA groups, although a trend toward improved post-operative symptom improvement was seen in the BMA group. Clinical outcomes are detailed in Table 2.

**Table 2:** Clinical outcomes. Clinical outcomes in 92 patients undergoing ACDF with – or without – the use of autogenous BMA. ACDF: Anterior cervical discectomy and fusion; BMA: Bone marrow aspirate.

	Total	BMA	Non-BMA	p
Mean Clinical Follow-up (Months)	22.5 ± 18.4	13.8 ± 8.4	31.3 ± 21.4	<0.001
Symptoms Improved Post-operatively	82/92 patients (89.1%)	49.52 patients (94.2%)	33/40 patients (82.5%)	0.096
Symptoms Stable Post-operatively	10/92 patients (10.9%)	3/52 patients (5.8%)	7/40 patients (17.5%)	0.096
Symptoms Worsened Post-operatively	0/250 patients (0%)	0/250 patients (0%)	0/250 patients (0%)	1
Minor Complications	4/92 patients (4.3%)	1/52 patients (1.9%)	3/40 patients (7.5%)	0.314
Reoperation at Same Level	8/92 patients (8.7%)	4/52 patients (7.7%)	4/40 patients (10%)	0.724
Osteostimulator Used Post-operatively	9/92 patients (9.8%)	4/52 patients (7.7%)	5/40 patients (12.5%)	0.495

Radiographic outcomes

**Figure 1:**
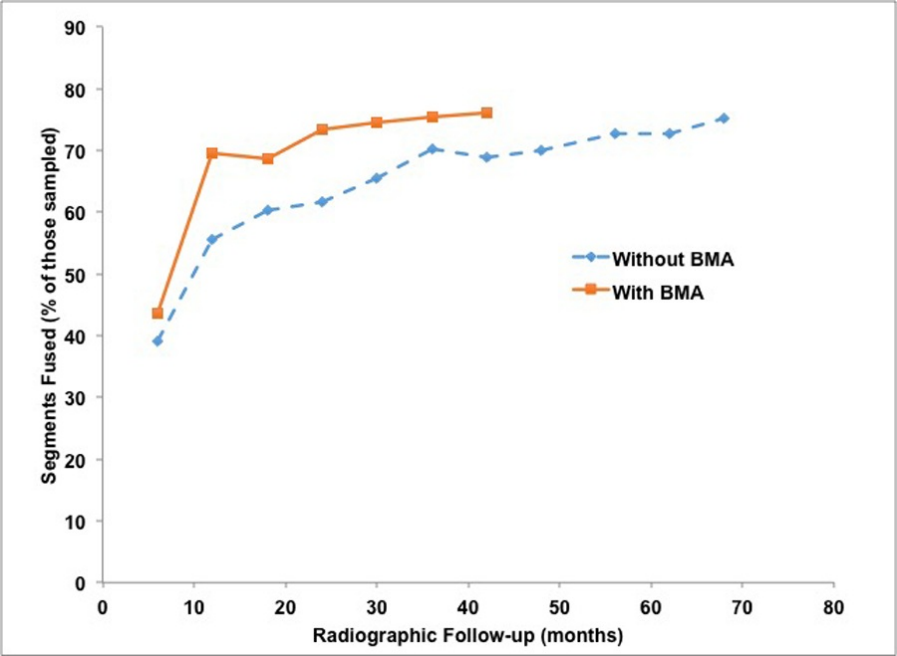
Fusion rates over time. Line graph illustration of radiographic fusion rates after ACDF with – or without – the use of autogenous BMA adjunct in the 92 study patients. Percentages are calculated for patients with available imaging data at each given time point in the follow-up period, as seen in Table 3. ACDF: Anterior cervical discectomy and fusion; BMA: Bone marrow aspirate.

**Figure 2:**
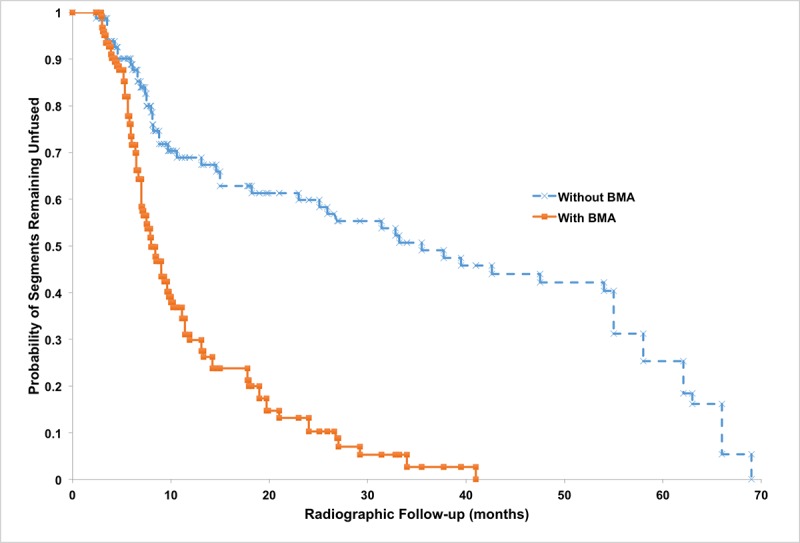
Probability of fusion over time. Kaplan-Meier survival analysis displaying probability of fusion over time for 286 instrumented segments treated with – or without – autogenous BMA. Log-rank test revealed a statistically significant difference between the two curves (p < 0.001). BMA: Bone marrow aspirate

The mean radiographic follow-up period for all 92 study patients was 21.4 ± 18.4 months. At the end of the study period, 154/203 (75.9%) of all instrumented segments were considered fused. No significant difference was seen between the fusion rates in the BMA versus the non-BMA group (P = 1, Table 3, Figure 1). The rate of fusion in the BMA group (9.8% of segments fused per month of follow-up), however, was significantly greater than that seen in the non-BMA group (6.1% of segments fused per month of follow-up, p = 0.003). Further investigation with Kaplan-Meier survival analysis and the log-rank test revealed that the probability of fusion over the course of radiographic follow-up was significantly greater for the BMA group when compared with the non-BMA group (p < 0.001; Figure 2). No significant relationship was seen between patient age (R = 0.232, p = 0.79), patient gender (p = 0.29), use of an osteostimulator (p = 0.85) or smoking status (p = 0.11) and the rate of fusion amongst study patients.

**Table 3:** Radiographic outcomes. Radiographic outcomes for 92 patients undergoing ACDF with – or without – the use of autogenous BMA. ACDF: Anterior cervical discectomy and fusion; BMA: Bone marrow aspirate.

	Total	BMA	Non-BMA	p
Mean Radiographic Follow-up (months)	21.4 ± 18.4	12.9 ± 8.3 (range, 6-41)	32.4 ± 21.8 (range, 6-66)	<0.001
Segments Fused at Latest Radiographic Follow-up	154/203 segments (75.9%)	93/122 segments (76.2%)	61/81 segments (75.3%)	1
6 months	40/94 segments (42.6%)	31/71 segments (43.6%)	9/23 segments (39.1%)	0.81
12 months	103/157 segments (65.6%)	78/112 segments (69.6%)	25/45 segments (55.6%)	0.098
18 months	110/166 segments (66.3%)	81/118 segments (68.6%)	29/48 segments (60.4%)	0.366
24 months	119/168 segments (70.8%)	88/120 segments (73.3%)	31/48 segments (61.6%)	0.266
30 months	125/174 segments (71.8%)	91/122 segments (75.4%)	38/54 segments (70.3%)	0.577
36 months	130/176 segments (73.9%)	92/122 segments (75.4%)	38/54 segments (70.3%)	0.577
42 months	133/180 segments (73.9%)	93/122 segments (76.2%)	40/58 segments (70.0%)	0.364
Segments Fused per Month of Radiographic Follow-up (%/month)	8.2	9.8	6.1	0.009

## Discussion

Rationale for use of BMA in spinal fusion

Successful arthrodesis relies upon the principles of osteogenesis, osteoconduction and osteoinduction [12]. Osteogenic potential is the capacity for a graft or other material to form new bone, a process dependent on the presence within a given site of an adequate number of viable cells with bone-forming capabilities (e.g., osteoprogenitor cells). Osteoconductiveness is the ability of a graft or other material to provide a suitable physical matrix for the influx and attachment of osteoprogenitor cells, neovascularization and bony deposition. Osteoinduction is the process whereby immature, pluripotent stem cells are induced by a variety of growth factors and other environmental cues to become mature osteoblasts, osteoclasts and osteocytes. ICBG and other autograft bone is said to possess all three of these qualities [2], but ICBG harvest leads to chronic donor site pain and even functional impairment in many patients [4,13]. Though PEEK possesses certain structural qualities desirable in an interbody graft (e.g., elastic modulus similar to that of bone; radiolucency allowing for post-operative visualization of bone growth) [14], being a synthetic polymer it does not itself contain osteogenetic, osteoconductive or osteoinductive properties. Instead, PEEK cages, like other synthetic cages, are typically combined with local bone and/or allograft materials (e.g., demineralized bone matrix [DBM]) possessing the qualities necessary to induce bone formation.

The capacity for bone marrow to induce ectopic bone formation was recognized as early as the 19th century [11]. Further study has suggested that this bone-forming ability is related to the presence within BMA of osteoprogenitor cells with inherent osteogenetic potential (albeit at a relatively low concentration [1/100,000-500,000 cells]) [11, 15, 16]. The presence of these osteoprogenitor cells within BMA, the relative ease with which BMA may be harvested, the low cost of BMA harvest and the low patient morbidity associated with harvest makes BMA an attractive adjunct to spinal fusion procedures. Indeed, numerous animal studies have demonstrated that BMA enhances the rate of spinal fusion seen with ICBG [17] or DBM [18-19]. Likewise, the results of some animal studies suggest that autologous BMA improves the fusion rates seen with engineered, biodegradable, osteoconductive matrices (e.g., CHA sponges) [20-22] and recombinant human bone morphogenetic protein (rhBMP) [23].

Literature evidence for BMA efficacy in spinal fusion

Very few clinical studies have evaluated the comparative efficacy of autologous BMA versus other graft materials in human spinal fusion [11]. Price et al. compared a BMA-DBM composite to ICBG and freeze-dried corticocancellous autografts in a retrospective series of 73 patients undergoing fusion for adolescent idiopathic scoliosis and found no significant difference between the pseudarthrosis rates of ICBG and BMA-DBM [24]. Korovessis et al. prospectively compared ICBG to hydroxyapatite + local bone + BMA in 57 patients undergoing decompression and posterolateral fusion for degenerative lumbar stenosis and found no difference in the fusion rates or clinical outcomes one-year postoperatively [25]. With respect to ACDF, specifically, no comparative studies are available. Papavero et al. prospectively evaluated 78 patients undergoing ACDF with a titanium cage filled with a hydroxyapatite/BMA mixture and favorable clinical and radiographic outcomes were seen, but no control (i.e., non-BMA) group was studied for comparison [10]. Similarly, Khoueir et al. retrospectively studied 66 patients who underwent ACDF with a combination of a fibular strut allograft, a CAH sponge and autogenous BMA [8]. Although favorable clinical and radiographic outcomes were demonstrated, no control group was available for comparison. The present series constitutes the first controlled study to evaluate the efficacy of a BMA-hydroxyapatite-PEEK-local bone combination versus hydroxyapatite-PEEK-local bone alone in ACDF.

The results of this study demonstrate that autogenous BMA, when combined with a CAH sponge, PEEK interbody graft and local autograft in the setting of ACDF, results in faster radiographic fusion than CAH/PEEK/local autograft alone. Although a trend was seen towards improved clinical outcomes and 12-month fusion rates in the BMA group, these results did not reach statistical significance.

Limitations                                                                                           

This study is limited primarily by its retrospective nature, which predisposes the results to errors of bias and confounding. In retrospective cohort studies comparing treatment results, selection bias is of particular concern. In the present study, the decision to treat with – or without – BMA was incidental and related only to the time during the study period at which the patient underwent ACDF, as the senior author did not use BMA as an adjunct to ACDF initially within the study period, but began to do so semi-exclusively in 2013 as a result of a growing body of evidence (described above) supporting the efficacy of BMA as an adjunct to ACDF. Similarly, the significant discrepancy between the length of radiographic follow-up between BMA and non-BMA groups was a factor of the same change in practice patterns. Patients undergoing ACDF without BMA were treated earlier in the study period than those undergoing ACDF with BMA.

An additional limitation inherent within studies of spinal fusion is the indirect assessment of spinal fusion through radiographic means, which has been shown to be unreliable at times [25,26]. The “gold standard” determination of spinal fusion occurs through operative exploration, but – with the exception of revision cases – this approach is not practical in a clinical setting. Both CT and static radiographs have been shown to overestimate fusion rates in some studies [26,27]. Dynamic radiographs are limited by extent of patient effort/motion between flexion and extension, measurement reliability, controversy regarding the degree of allowable motion and the problematic endeavor of using two-dimensional films to evaluate a three-dimensional process (i.e., fusion) [27]. In an effort to provide greater specificity regarding fusion rates in this study (perhaps at the expense of sensitivity), we utilized dynamic films and/or CT imaging alone in our determination of fusion rates, and the criteria employed were a relatively strict combination of those employed throughout the literature [28-30].

Additional investigation is needed to determine to what degree altering the concentration or viability of osteoprogenitor cells present within BMA (e.g., through centrifugation or culturing) influences radiographic and/or clinical outcomes of spinal fusion, as some authors have suggested [6,19].

## Conclusions

BMA is a readily accessible, low-cost adjunct to ACDF that enhances the fusion rates seen with a CAH/PEEK/local autograft combination. Further investigation is warranted regarding the value of BMA post-processing and the combination of BMA with other grafting materials in ACDF.
